# Confocal Laser Endomicroscopy Can Improve the Diagnosis Rate and Range Assessment of Patients With Conflicting Chronic Atrophic Gastritis Results of White Light Endoscopic and Pathological Diagnosis

**DOI:** 10.3389/fonc.2022.809822

**Published:** 2022-03-24

**Authors:** Suya Pang, Hailing Yao, Chen Jiang, Qin Zhang, Rong Lin

**Affiliations:** ^1^Department of Gastroenterology, Union Hospital, Tongji Medical College, Huazhong University of Science and Technology, Wuhan, China; ^2^Department of Pathology, Union Hospital, Tongji Medical College, Huazhong University of Science and Technology, Wuhan, China

**Keywords:** chronic atrophic gastritis, gastric cancer, white light endoscopy, confocal laser endomicroscopy, pathology

## Abstract

**Background and Aims:**

Chronic atrophic gastritis (CAG) is closely related to the development of gastric cancer. However, the diagnostic accuracy of white light endoscopy (WLE) biopsy for CAG is poor. The diagnostic role and efficacy of confocal laser endomicroscopy (CLE) in CAG missed under WLE biopsy remain unclear.

**Methods:**

This study is a single-center prospective study that included 21 patients from 1,349 patients who underwent WLE and biopsy and whose WLE results confirmed CAG, but pathological results did not. Then, all these patients received CLE examination and underwent targeted biopsies and five-point standard biopsies. The sensitivity, specificity, and accuracy of CLE diagnosis and targeted biopsy were analyzed.

**Results:**

The pathological results of five-point standard biopsies in 21 patients confirmed CAG, and 17 patients (81.0%) were confirmed to have intestinal metaplasia (IM). According to the image diagnosis of CLE, there were 19 cases (90.5%) of CAG and 14 cases (66.7%) of IM among these 21 patients. According to the targeted biopsy of CLE, 17 cases (81.0%) of CAG and 14 cases (66.7%) of IM were diagnosed. There was no significant difference between CLE image diagnosis and five-point standard biopsies in terms of atrophy severity score (p = 0.927), IM severity score (p = 0.250), atrophy scope score (p = 0.781), and IM scope score (p = 0.195). For CAG, the sensitivity and accuracy of CLE image diagnosis were higher than those of CLE targeted biopsies (90.5% vs. 81.0%, p = 0.331), but for IM, the diagnosis was the same.

**Conclusions:**

CLE can improve the diagnosis rate of CAG and can increase the comprehensive assessment of the scope and severity of CAG.

## Introduction

As the fourth most common cancer worldwide, gastric cancer has great significance in prevention and treatment (1). Chronic atrophic gastritis (CAG) is a well-recognized precancerous lesion of gastric cancer and its diagnosis and follow-up are critical (2, 3). The most common diagnostic and follow-up method of CAG currently is white light endoscopy (WLE) and endoscopic biopsy.

However, WLE cannot accurately diagnose CAG, and the WLE diagnosis has two limitations. Firstly, WLE cannot accurately locate the lesion, resulting in targeted biopsy failure. Therefore, there may be inconsistencies between WLE diagnosis and pathological diagnosis (4). A national multicenter study in China found that the sensitivity of WLE to diagnose CAG was less than 50%, and most specificities and positive predictive values were less than 70% (5). Secondly, local biopsy under WLE not only cannot accurately reflect the severity of the lesion because it cannot achieve targeted biopsy but also cannot assess the extent of the lesion. However, the degree and scope of atrophy affect its prognosis (6, 7).

Confocal laser endomicroscopy (CLE) stands at the forefront of the novel endoscopic techniques. CLE permits direct *in vivo* identification and clear visualization of gastric patterns at the cellular level (8, 9), which makes it assess the severity and scope of atrophy effectively. Several studies reported that CLE is useful in the diagnosis of cancer and premalignant lesions (10–12). Furthermore, some studies showed that CLE had greater consistency with the pathological diagnosis result than WLE (12, 13).

Actually, a great number of patients had the CAG diagnosis confirmed with WLE and the chronic inflammation diagnosis confirmed with histopathology clinically (4), which brings difficulty in final diagnosis and treatment for clinicians. Therefore, in this study, we aim to use CLE to diagnose those patients who were diagnosed with CAG under WLE, and whose pathological results were chronic inflammation, to explore the diagnostic value of CLE for CAG patients.

## Methods

### Patients

We selected subjects from 1,349 patients with both biopsy and pathological diagnoses among 6,258 patients who underwent WLE in the Union Hospital of Tongji Medical College of Huazhong University of Science and Technology. Among them, 21 patients were included in this study according to the inclusion and exclusion criteria. Inclusion criteria were as follows: 1) 18–80 years old, can endure digestive endoscopy; 2) patients with WLE diagnosis of CAG, and pathological results were chronic inflammation in nearly 3 months; and 3) voluntarily joined the experiment and signed written informed consent. Exclusion criteria were as follows: 1) patients with gastrectomy history, acute gastrointestinal bleeding history, advanced gastric cancer, and other gastrointestinal malignancies; 2) patients suffering from disease who cannot tolerate gastroscopy, such as coagulation dysfunction, renal insufficiency, serious cardiopulmonary disease, and sodium fluorescein allergy; 3) pregnant and lactating women; and 4) no legal capacity or having medical and ethical reasons that affect the study. All patients included in this study signed an informed consent form.

### Confocal Laser Endomicroscopy Procedure

All patients were given oral saline solution containing simethicone emulsion (Berlin-Chemie AG) to remove excess mucus from gastric mucosa before examination. And all patients underwent a skin test of fluorescein sodium (Baiyunshan Mingxing Pharmaceutical Co. Ltd., Guangzhou, China) before the examination to ensure that allergic reactions did not occur, which was used as a contrast agent intravenously before CLE.

First, the condition of the mucosa was observed through WLE, then eroded, elevated, hyperemic, and rough or granular mucosa and gray intestinal-type epithelium were selected as targets, then CLE was used for observation (14). CLE (YZB/FRA 5399-2012) was performed by an experienced endoscopist whose endoscopy experience was the same as that of WLE endoscopists. Five standardized intragastric sites were observed on CLE: lesser curvature of the antrum, greater curvature of the antrum, stomach angle, lesser curvature of the corpus, and greater curvature of the corpus. Meanwhile, 5–10 CLE images of different mucosal depths and CLE video were collected from each site, and the images and videos were stored for later assessment. The endoscopist performed real-time diagnosis, targeted biopsy, and five standard site biopsies under CLE. And another experienced endoscopist made diagnoses through images and videos and was blinded to the basic information and medical history of patients.

**Figure 1:**
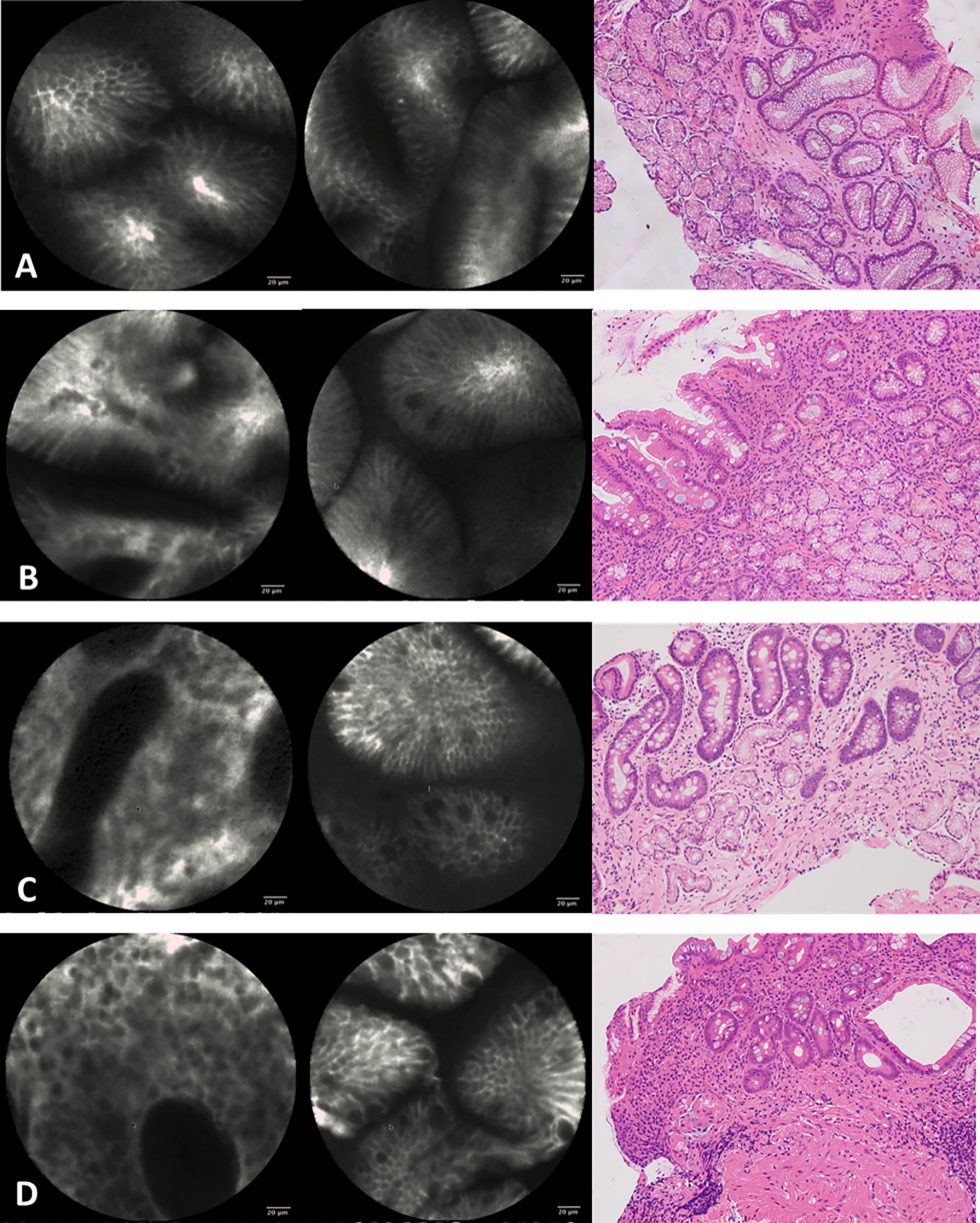
Confocal imaging and histopathology of gastric epithelium. **(A)** Normal gastric epithelium. **(B)** Mild atrophic gastritis and intestinal metaplasia. **(C)** Moderate atrophic gastritis and intestinal metaplasia. **(D)** Severe atrophic gastritis and intestinal metaplasia.

The standard criteria for endoscopists to diagnose CAG with CLE are that stomach pits are sparse and there are interstitial widening, irregular arrangement, severely reduced number of gastric pits, dilated openings, and a decreased number of subepithelial capillaries (15). The criteria for diagnosing IM with CLE are goblet cells, villous‐like pits, absorptive cells, and brush borders (16) (**Figure 1**).

### Histopathology

Mucosal biopsy specimens were placed in 4% “methanal” or “formaldehyde” sectioned into 4-μm thickness, and stained with hematoxylin and eosin (HE). Two experienced pathologists reviewed all the biopsy specimens of targeted biopsy under WLE and CLE. Meanwhile, they were blinded to both endoscopy information and results and the medical history of patients, and they were unaware of each other’s diagnoses. CAG was recognized morphologically by mild epithelial abnormalities, including a decrease in the number of cytoplasmic mucins, an increase in the nucleus and nucleolus, and an increase in mitotic figures in the pits of the stomach, when inflammation was extensive and accompanied by glandular atrophy (17). And the CAG was further divided into mild, moderate, and severe by a rough assessment of the relationship between the thickness of the gland and the thickness of the entire mucosa. IM was recognized morphologically by goblet cells and absorptive cells and was further divided into mild, moderate, and severe according to the number of abnormal cells (18) (**Figure 1**).

### Study Definitions

Atrophy range score was a diagnosis of CLE and five standard site biopsy pathologies for the scope of atrophy. The diagnosis results of different parts of the stomach with or without atrophy were recorded as 1 point and 0 points, respectively. Atrophy severe score was a diagnosis to evaluate the severity of atrophy by CLE and five standard site biopsy pathologies. Diagnosis of different parts of the stomach as mild to moderate atrophy was recorded as 1 point, 2 points, and 3 points. IM range score and IM severe score were defined in the same way.

### Statistical Analysis

Data were entered into Excel data sheets and analyzed with SPSS software v25.0 (IBM, USA) or SAS software, version 9.4. Clinical characteristics were expressed as median and range, absolute value or fractions. Patients characteristics were compared using χ^2^ or Fisher exact test for categorical variables. A Mann–Whitney U-test was used to compare the median duration between groups. p < 0.05 was considered statistically significant.

## Results

### Patient Characteristics

We included 21 patients with WLE diagnosis of CAG and chronic inflammation on pathological examination for nearly 3 months in this prospective study. The overall median age was 54 years (range: 27–66 years), with a women-to-men ratio of 1.33. Fourteen (66.7%) patients had a history of proton pump inhibitor (PPI) medication. Eight (38.1%) patients had a history of smoking and drinking. And 8 (38.1%) patients were anxious, and 3 (14.3%) patients were depressed. The demographic characteristics of patients identified in this study are summarized in **Table 1**.

**Table 1:** Patient demographics and clinical features in this study.

Characteristics	All patients (n = 21, %)
**Mean age, years (range)**	54 (27–66)
**Sex**	
** Male**	9 (42.9)
** Female**	12 (57.1)
**History of PPI use**	14 (66.7)
**Smoking habits**	8 (38.1)
**Drinking habits**	8 (38.1)
**Anxiety**	8 (38.1)
**Depression**	3 (14.3)

PPI, proton pump inhibitor.

### Confocal Laser Endomicroscopy Results and Pathological Diagnosis Results

Among the 21 patients, pathological results of five standard site biopsies all confirmed CAG, and 17 (81.0%) of them had IM. The real-time diagnosis results through CLE and the post-diagnosis results through images and videos were consistent. And the diagnoses of the two pathologists were also consistent. Among the 21 patients, 19 (90.5%) patients were diagnosed with CAG by CLE, and 14 (66.7%) patients were diagnosed with IM. In the case of targeted biopsy, 17 (81.0%) patients were diagnosed with CAG, and 14 (66.7%) patients had IM.

Most patients had CAG and IM in multiple sites. Compared with targeted biopsy, CLE image diagnosis was more consistent with five-point standard biopsies in the diagnosis of CAG and IM. There was no significant difference between the CLE diagnosis and the pathological diagnosis of five standard site biopsies for the atrophy range score and atrophy severe score (p = 0.781; p = 0.927) and also for the IM range score and IM severe score (p = 0.195; p = 0.250). The results of confocal diagnosis, targeted biopsy, and five standard site biopsies are shown in **Table 2**.

**Table 2:** The diagnosis results of CLE image diagnosis, CLE targeted biopsies, and five-point standard biopsies.

	CLE diagnosis	Targeted biopsy	Five standard site biopsies	p	p
**Chronic inflammation**	2	4	0	0.488	0.027
**CAG**	19	17	21	0.488	0.027
**Antrum**	3	9	5	0.697	0.012
**Angle**	3	2	0	0.106	0.139
**Body**	0	0	1	1.000	0.618
**Multiple parts**	13	6	15	0.731	0.026
**Atrophy score**					
**Range**	46	–	48	0.781	–
**Severe**	80	-	79	0.927	-
**IM**	14	14	17	0.484	0.242
**Antrum**	1	4	3	0.607	0.077
**Angle**	2	0	2	1.000	0.493
**Body**	0	0	0	-	-
**Multiple parts**	11	10	12	0.698	0.637
**IM score**					
**Range**	33	–	42	0.195	–
**Severe**	64	-	76	0.250	-

CLE, confocal laser endomicroscopy; CAG, chronic atrophic gastritis; IM, intestinal metaplasia.

The sensitivity and accuracy of the CLE image in the diagnosis of CAG were both 90.5% and of CLE targeted biopsy were both 81.0%. For CAG, CLE image diagnosis had higher sensitivity and accuracy than CLE targeted biopsies but not statistically significant (p = 0.331), but for IM, they were the same. The sensitivities of the CLE image and CLE targeted biopsy in the diagnosis of IM were both 76.5%, the specificity was 75%, and the accuracy was 76.2%. The results of diagnostic efficiency of CLE and targeted biopsy on CAG and IM are shown in **Table 3**.

**Table 3:** Diagnostic efficiency of CLE and targeted biopsy on CAG and IM.

	CAG			IM		
	Sensitivity	Specificity	Accuracy	Sensitivity	Specificity	Accuracy
**CLE**	90.5%	–	90.5%	76.5%	75%	76.2%
**Targeted biopsy**	81.0%	-	81.0%	76.5%	75%	76.2%
**p**	0.331	–	0.331	0.672	0.672	0.672

CLE, confocal laser endomicroscopy; CAG, chronic atrophic gastritis; IM, intestinal metaplasia.

## Discussion

Each year, there are more than 900,000 new diagnoses of gastric cancer worldwide, and gastric cancer is the second and fourth most common cause of cancer death in men and women, respectively (19). As a precancerous lesion of gastric cancer, CAG is of great significance in diagnosis. There was a great number of patients with inconsistent results of WLE and pathological diagnosis of CAG clinically (4), which made difficulties in clinical diagnosis and treatment. But there were few studies for the diagnosis of these contradictory patients. CLE can realize real-time diagnosis at the cellular level and a larger evaluated area *in vivo* identification compared to biopsies (8, 9). And many studies have shown that CLE has a high diagnostic value for precancerous lesions (20, 21), but few studies had used CLE to diagnose patients with inconsistent WLE diagnosis and pathology diagnosis. Therefore, this paper made a prospective study of these patients with conflicting diagnosis results and made diagnoses of CAG and IM by CLE, targeted biopsy, and five standard site biopsies that were regarded as the gold standard, so as to evaluate the diagnostic efficacy of CLE for CAG patients.

We studied 21 patients with conflicting diagnosis results, then all patients were diagnosed as CAG by the gold standard method, and 90.5% of patients were diagnosed with CAG through CLE. The sensitivity of CLE diagnosis of atrophy in the study was as high as in other studies (ranging from 85% to 90%) (12, 15, 22–29). Besides, targeted biopsy under CLE also diagnosed 81.0% of patients with CAG, which indicated that CLE can improve biopsy accuracy. As a result, CLE can improve the diagnosis rate of CAG. However, the accuracy of CAG diagnosis by targeted biopsy was lower than that of CLE. This may be because the biopsy under CLE can only be performed after the confocal probe was removed, and there may be displacement during this process. If there is a device that can simultaneously perform a biopsy in the CLE magnified field of view, this problem may be solved. Furthermore, CLE did not differ significantly from the gold standard method in assessing the scope and severity of atrophy and IM. This shows that CLE can achieve a comprehensive assessment of the scope and severity of CAG and IM compared with WLE.

In conclusion, experienced endoscopists can use WLE or CLE to diagnose CAG effectively, but the consistency of WLE diagnosis and pathological diagnosis of biopsy needs to be improved. This study shows that CLE can solve the contradiction between WLE and pathological diagnosis and increase the diagnostic rate of patients with different WLE and pathological diagnoses. CLE can achieve targeted biopsy, and as a real-time pathology, the diagnostic efficiency was even higher than that of targeted biopsy. The most important point was that CLE had a good advantage in the diagnosis, follow-up, and comprehensive evaluation of CAG, and it was significantly better than WLE and biopsy under WLE. CLE had good clinical significance for the diagnosis of CAG.

## Data Availability Statement

The original contributions presented in the study are included in the article/supplementary material. Further inquiries can be directed to the corresponding author.

## Ethics Statement

The studies involving human participants were reviewed and approved by the Ethics Committee of Tongji Medical College, Huazhong University of Science and Technology (IORG No: IORG0003571). The patients/participants provided their written informed consent to participate in this study.

## Author Contributions

RL designed and supervised the study and data analysis. SP performed most of the investigation and data analysis and wrote the manuscript. HY and CJ contributed to interpretation of the data and analyses. QZ provided pathological diagnosis assistance. All authors contributed to the article and approved the submitted version.

## Funding

This study was supported by the National Natural Science Foundation of China (nos. 81770539, 81974068, and 81900580).

## Conflict of Interest

The authors declare that the research was conducted in the absence of any commercial or financial relationships that could be construed as a potential conflict of interest.

## Publisher’s Note

All claims expressed in this article are solely those of the authors and do not necessarily represent those of their affiliated organizations, or those of the publisher, the editors and the reviewers. Any product that may be evaluated in this article, or claim that may be made by its manufacturer, is not guaranteed or endorsed by the publisher.

## Abbreviations

CAG, chronic atrophic gastritis; WLE, white light endoscopy; CLE, confocal laser endomicroscopy; IM, intestinal metaplasia.
